# Stem cells as biological drugs for incurable diseases

**DOI:** 10.1186/s13287-025-04632-8

**Published:** 2025-10-08

**Authors:** Mahmood S. Choudhery, Akihiro Umezawa

**Affiliations:** 1 Department of Human Genetics and Molecular Biology, University of Health Sciences Lahore, Lahore, 54000 Pakistan; 2 Center for Regenerative Medicine, National Center for Child Health and Development Research Institute, Tokyo, Japan

Today – October 8, 2025, marks *Stem Cell Awareness Day* – a significant occasion proudly celebrated by the Editorial Board of *Stem Cell Research & Therapy*. This Editorial, authored by members of our Board, highlights the critical importance of stem cell research and its broad impact across medical disciplines.

Medical fields not formally labeled as “cell therapy” share the same underlying principle. Hematopoietic stem cell transplantation (HSCT) stands as the prototypical success of cell therapy. Its effectiveness rests on the remarkable ability of donor-derived stem cells to engraft, self-renew, and reconstitute the immune and hematopoietic systems after intensive conditioning. Likewise, reproductive medicine relies on transferring a totipotent fertilized egg into the maternal uterus to establish pregnancy. This represents a form of cellular transplantation harnessing maximal developmental potential. In addition, epidermal cell sheets represent one of the oldest and most widespread applications of regenerative medicine. In burns, vitiligo, genetic diseases, and trauma care, autologous or allogeneic epidermal cell sheets provide wound coverage, reduce infection, and accelerate healing. Their success depends on cell engraftment, proliferation, and integration with host tissue. Decades of clinical experience affirm both their potential and the necessity of strict manufacturing standards, consistent quality control, and long-term safety monitoring. Across these diverse examples, one unifying principle emerges: the success of cellular therapies relies on recapitulating or leveraging the intrinsic behavior of stem cells within the body—engraftment, proliferation, and differentiation. More recently, transplantation of stem cells has also been seen to have therapeutic efficacy via paracrine mechanisms of action and by modulation of inflammation and the immune system. Whether rebuilding immunity via HSCT, eradicating cancer with CAR-T cells, regenerating skin with keratinocytes, establishing pregnancy with embryos, or pursuing new strategies such as hepatocyte progenitor transplantation for liver failure, the common mechanism remains the same. Future advances must address hurdles in safety, scalability, immune management, and manufacturing to fully realize the transformative promise of regenerative medicine.

“Stem cell research and therapy” offers new hope for patients suffering from diseases that cannot be cured with conventional medicine. Neurodegenerative disorders (like Alzheimer’s and Parkinson’s disease), chronic organ failures (such as heart failure and liver cirrhosis), complex autoimmune disorders (such as multiple sclerosis, scleroderma), pediatric conditions (such as cerebral palsy, autism spectrum disorder, spinal muscular atrophy), age-related disabilities (such as osteoarthritis), and certain genetic disorders are among the incurable diseases. These diseases impose persistent challenges for health care system worldwide. Additionally, these diseases place a significant emotional, physical and financial burden on patients and their families. Existing medical approaches only focus on the management of symptoms and slow disease progression through conventional medication. These approaches, however, rarely reverse the disease process and often provide only temporary relief, leaving patients and their families still searching for better options. With a global aging demographic, these chronic conditions are becoming even more common, adding to the pressure on individuals and healthcare systems alike. In response to this growing challenge, stem cell-based therapy and regenerative medicine has emerged as one of the most promising next-generation approaches in contemporary medicine. Stem cell-based therapies offer the potential to repair, replace, or regenerate damaged tissues and restore normal physiological function at the cellular level [1].

Stem cells possess two defining characteristics, i.e. self-renewal (the ability to divide and produce identical copies of themselves) and differentiation (the ability to differentiate into specialized cell types). These characteristics make stem cells promising candidates for the repair and regeneration of damaged tissues and organs. The self-renewal as well as differentiation potential is, however, not the same for all types of stem cells, and depends largely on two factors: (1) their source, and (2) their stage of development. Embryonic stem cells (ESCs) are pluripotent, which means that they can differentiate into all types of cells in the body, including those of the ectoderm (e.g., skin and neurons), mesoderm (e.g., muscle and blood), and endoderm (e.g., liver and pancreas) [2]. This makes ESCs the most promising for research and therapeutic applications; however, their use is associated with serious ethical, regulatory and safety concerns. Adult (or somatic) stem cells, such as hematopoietic stem cells (HSCs, found in bone marrow) and mesenchymal stem cells (MSCs, found in various adult tissues such as bone marrow, adipose tissue, dental pulp, etc.), have a more limited proliferative and differentiation capacity. They are typically multipotent, meaning they can differentiate into a limited range of cell types related to their tissue of origin. Induced pluripotent stem cells (iPSCs) are adult cells that have been genetically reprogrammed to behave like ESCs. iPSCs, like ESCs, are also pluripotent and capable of giving rise to any cell type in the body, offering a promising alternative for personalized medicine without the ethical concerns associated with embryonic sources, although potential tumorigenicity concerns remain.

Due to their dynamic and adaptive therapeutic properties, stem cells are considered “living drugs”. Unlike conventional medicines, which are typically derived from chemical or biological compounds, living drugs such as stem cells are derived from living tissues and are administered to patients as viable and functional cells. The effects of conventional medicine (if available for a disease) are often temporary, whereas living drugs can become part of the damaged tissues and organs, exerting longer-lasting effects. Additionally, conventional drugs must be administered repeatedly, while a single dose of a living drug may have a profound and sustained impact. The life cycle of a conventional drug includes absorption (entry into blood stream), distribution (transport to target tissue), metabolism (breakdown, typically in the liver) and excretion (elimination from body). However, living drugs are not readily excreted from the body. After transplantation, they tend to home to the injury site and integrate into the tissues where they actively contribute to the repair and regeneration of damaged body parts (Fig. 1), although in some cases, stem cells have been observed to not remain at the diseased tissue site and thus exert their therapeutic effects transiently or remotely via paracrine mechanisms [3].

**Fig. 1:**
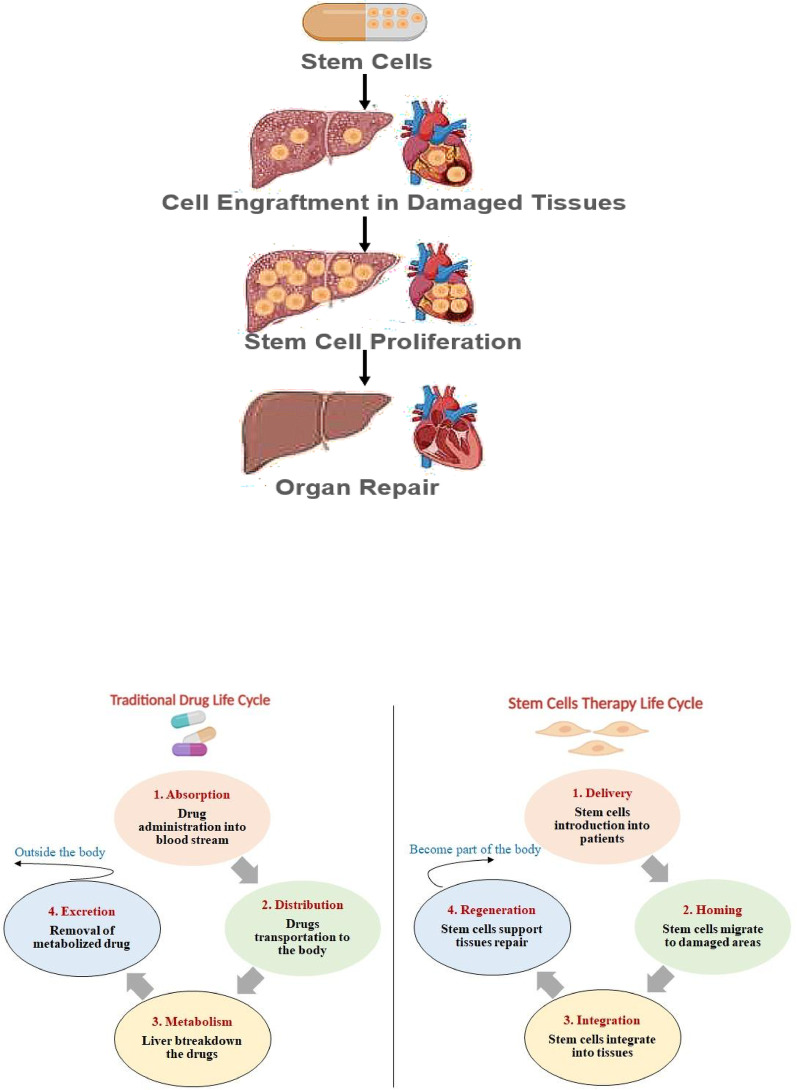
Stem cells as a living drug (top). A comparison between the life cycles of living drugs (like stem cells) and conventional drugs (chemical or biological compounds) (below). Traditional medicines have a defined life cycle that involves absorption into the bloodstream, distribution throughout the body, metabolism (often in the liver), and ultimately excretion from the body. On the other hand, living drugs like stem cells are administered as viable, functional cells. After entering the body, they tend to home to the site of injury and become a part of the damaged tissue. They regenerate the damaged organs and tissue by differentiating into specific cell types and by secreting bioactive molecules that promote long-term repair and regeneration

Stem cells have the unique ability to sense the signals from the injured and diseased tissues, respond to environmental cues, and adapt to the biological microenvironment in which they reside. Stem cells exert their therapeutic effects through a combination of mechanisms, including differentiation into specific cell types, paracrine signaling, immunomodulation, homing and migration to the injury sites, tissue integration as well as anti-apoptosis and anti-fibrotic actions [4]. Table 1 shows these key mechanisms, highlighting their primary functions along with relevant examples and clinical applications.

**Table 1:** Various mechanisms by which stem cells exert their therapeutic effects

Mechanism	Primary function	Example of diseases
Differentiation	Replace lost or damaged cells in organs	Parkinson’s, spinal cord injury, osteoarthritis
Paracrine signaling	Promote healing and repair through secreted factors	Heart failure, wound healing
Immunomodulation	Control autoimmune and inflammatory responses	Multiple sclerosis, Crohn’s disease
Homing & Migration	Travel to site of injury	Rheumatoid arthritis, stroke
Engraftment & Integration	Functional integration into tissue	Retinal diseases, diabetes
Anti-apoptotic & Anti-fibrotic	Reduce cell death and scarring	Liver disease, pulmonary fibrosis

Following are just a few examples of how stem cells have the potential to revolutionize the field of regenerative medicine by providing cure for currently incurable diseases. These examples use stem cells as experimental medicine as the approaches are currently under evaluation. Recent studies show that patients with multiple sclerosis who fail to respond to conventional therapy can be treated using HSC therapy. HSC transplantation in these patients reboots the immune system, halting disease progression or even reversing some neurological damage. The potential for stem cell therapy has also been explored to make insulin-producing β cells, potentially freeing diabetic patients from insulin injections. This treatment will potentially reduce or even eliminate the need for lifelong insulin injections. Interestingly, early results from clinical trials launched by some biotech companies show the efficacy of encapsulated β-cells in sustained insulin production. In cardiology, injections of stem cells to patients with heart failure are being studied to repair damaged myocardium that was once believed to be beyond repair. Studies have reported improvements in heart function, reduced scar size, and increased quality of life following such therapies. Exciting advances in clinical trials of spinal cord injury treatment using stem cells also have enormous potential. Stem cells derived from embryonic tissue or umbilical cord blood, are being used in clinical trials to help paralyzed patients regain some degree of movement. Progress is also being made in treating Parkinson’s disease, where pluripotent stem cell derived dopaminergic neurons are transplanted into the brains of patients. There is new hope for long-term illness management as early-phase trials in Europe and Japan have revealed positive evidence of dopaminergic repair and motor improvement. Stem cells are also being explored in the repair of damaged retinal pigment epithelium in the treatment of age-related macular degeneration. Additionally, researchers are developing liver organoids using stem cells for application in the treatment of patients with end-stage liver failure to possibly lessen their reliance on liver transplants. MSC injections are also being tested in the treatment of the age-associated degenerative joint disease, osteoarthritis, to lessen inflammation and promote cartilage regeneration, providing a potential alternative to invasive joint replacement surgery. These early outcomes of stem cell based therapeutic trials are not simply encouraging but have the potential to be revolutionary, even if there is still more to be done and not all stem cell therapies have fully entered clinical use [5]. Figure 2 shows that stem cells are emerging as intelligent therapeutic agents, demonstrating the ability to adapt and respond to changes within their microenvironment. The dynamic responsiveness to local cues allows stem cells to play a key role in targeted therapies and tissue regeneration.

**Fig. 2:**
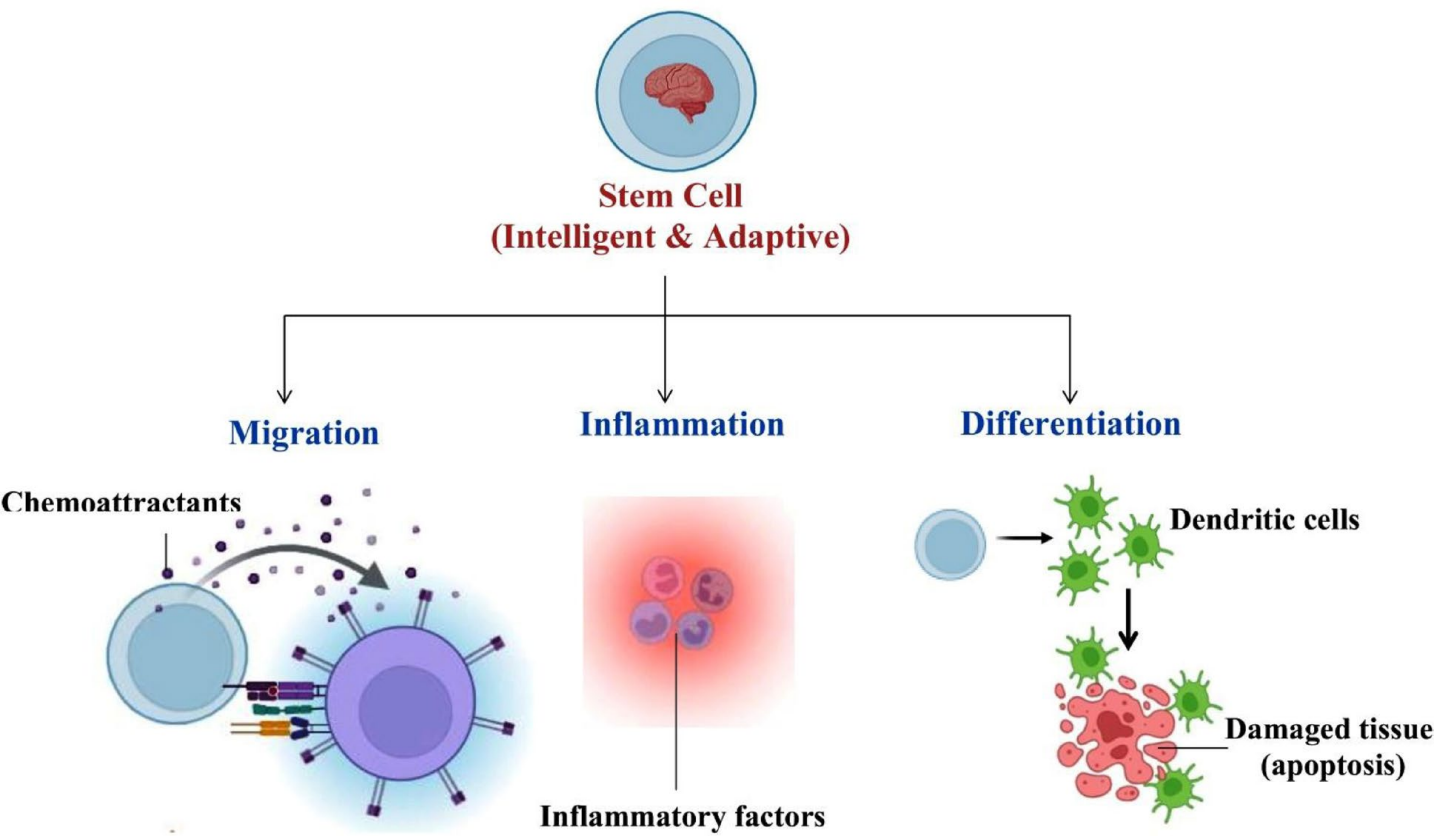
Stem cells as intelligent therapeutic agents, capable of dynamically responding to their microenvironment

Interestingly, other approaches, although not traditionally classified as " (stem) cell therapy” in regulatory contexts, are operated on the same biological principles, i.e. survival, proliferation, integration and proper function of (stem or progenitor) cells. One prominent example is reproductive medicine where fertilized embryos (totipotent stem cells) are implanted to initiate pregnancy. Similarly, in dermatology and burn care, skin grafting and cultured epidermal cell sheets represent the earliest and most widespread regenerative medicine applications. Other examples include bone marrow transplantation, widely used to restore immune function after chemotherapy or in genetic blood disorders; CAR-T cell therapy, where genetically engineered T-cells are designed to identify and destroy cancer cells; adipose tissue in different forms (such as macrofat, microfat, nanofat etc.) in aesthetic and reconstructive medicine; corneal transplantation for restoration of eyesight; and ACI (autologous chondrocyte implantation), which uses the patient’s own chondrocytes (cartilage cells) for cartilage repair in procedure that which uses the patient’s own chondrocytes (cartilage cells) to fix damaged knee cartilage etc.

The data from preclinical and clinical studies show that stem cell therapies are promising and can improve function and quality of patient life. These findings support the idea of the use of stem cells as “living drugs.” However, stem cell applications as living drugs are not without limitations and pose challenges such as ethical concerns, regulatory hurdles, immune rejection, risks like uncontrolled cell growth, standardization, delivery methods and long-term safety [6]. The functional properties of stem cells can also vary significantly depending on the tissue source, donor age and health status as well as protocols used for their production. These differences compromise the potency, consistency, clinical reproducibility and standardization of cell-based therapy. iPSC technology as well as other evolving regulatory frameworks offer some hope; however, issues like high costs, complex manufacturing, potential tumorigenicity, and limited accessibility remain to be resolved. Additional challenges include stem cell delivery routes and survival of transplanted cells. To cope with the hostile biological environment of injured or diseased tissue, strategies to enhance the survival of cells, and their homing and integration into appropriate tissues will need to be optimized.

The increasing problem of quackery and false information about stem cell treatments is another significant issue that needs to be addressed. Some unregulated facilities and so-called stem cell experts are taking advantage of vulnerable patients. They are not only charging outrageous prices and making misleading claims of efficacy but also offer untested treatments which give false hope. These unethical practices cast a dark shadow over the entire field by putting patients in danger as well as undermining public confidence. Consequently, they add to the increasing suspicion and negativity over what is otherwise a promising field of medicine with the potential to be transformative.

Stem cell-based treatments raise concerns as well as hope because it is difficult to make accurate conclusions regarding safety or efficacy, due primarily to the lack of standardization procedures from data collection to outcome evaluation. Access to stem cell therapies is globally unequal, which presents another challenge. While wealthier nations are at the forefront of discovery and clinical applications, low- and middle-income countries have challenges in infrastructure, knowledge, and regulatory capacity. The implementation of equitable distribution strategies and international collaborations is essential to ensure equity. Notwithstanding these challenges, stem cells represent a promising area of contemporary medicine. The list of diseases that once believed to be “incurable” is getting shorter with every new discovery in the field.

In conclusion, although much research and discovery remain, the rapidly emerging findings of stem cell therapy are not just hopeful but are revolutionary. Not only do stem cells have the potential to extend and improve the quality of life, but they have completely altered our knowledge, approach and potential for making decisions about disease treatment. The idea of stem cells as “living drugs” has shifted the focus from chemical drugs to living cells. Instead of just managing symptoms, stem cells represent potentially disease-modifying therapies that aim to actively repair and regenerate tissues. Stem cell research and therapy is therefore the future of personalized, targeted and potentially curative treatments. The word “incurable” was once considered synonymous with dejection. Better understanding and advancements in the field of stem cells will potentially shorten the list of incurable diseases. However, innovation and responsibility must be aligned in order to turn this promise into actual reality. It is imperative that we not only celebrate achievements on this Stem Cell Awareness Day, but also reinforce our shared dedication to ethical stewardship, technological innovation, regulatory harmonization, and scientific rigor, so that every person in all corners of the world can share the benefits of stem cell therapy.

## References

[1] Deng S, Xie H, Xie B. Cell-based regenerative and rejuvenation strategies for treating neurodegenerative diseases. Stem Cell Res Ther. 2025;16(1):167. 10.1186/s13287-025-04285-7. 40189500 10.1186/s13287-025-04285-7 PMC11974143

[2] Umezawa A, Fukuda A, Horikawa R, Uchida H, Enosawa S, Oishi Y, Nakamura N, Sasaki K, Yanagi Y, Shimizu S, Nakao T, Kodama T, Sakamoto S, Hayakawa I, Akiyama S, Saku N, Miyata S, Ite K, Javaregowda PK, Toyoda M, Nonaka H, Nakamura K, Ito Y, Fukuhara Y, Miyazaki O, Nosaka S, Nakabayashi K, Haga C, Yoshioka T, Masuda A, Ohkura T, Yamazaki-Inoue M, Machida M, Abutani-Sakamoto R, Miyajima S, Akutsu H, Matsubara Y, Igarashi T, Kasahara M. First-in-human clinical study of an embryonic stem cell product for urea cycle disorders. Stem Cell Res Ther. 2025;16(1):120. 10.1186/s13287-025-04162-3. PMID: 40050977. 40050977 10.1186/s13287-025-04162-3 PMC11887382

[3] Fujino A, Fuchimoto Y, Mori T, Kano M, Yamada Y, Ohno M, Baba Y, Isogawa N, Arai K, Yoshioka T, Abe M, Kanai N, Takagi R, Maeda M, Umezawa A. Evaluation of safety and efficacy of autologous oral mucosa-derived epithelial cell sheet transplantation for prevention of anastomotic restenosis in congenital esophageal atresia and congenital esophageal stenosis. Stem Cell Res Ther. 2023;14(1):86. 10.1186/s13287-023-03321-8. PMID: 37055850. 37055850 10.1186/s13287-023-03321-8 PMC10099682

[4] Ma C, Yu A, He T, Qian Y, Hu M. Stem cell therapy approaches for ischemia: assessing current innovations and future directions. Int J Mol Sci. 2025;26(13):6320. 10.3390/ijms26136320. 40650096 10.3390/ijms26136320 PMC12250106

[5] Jiang S, Bao X, Zhong C, Wang R. Mapping the global clinical landscape of stem cell therapies for neurological diseases from 1998 to 2023: an analysis based on the trialtrove database. Stem Cell Res Ther. 2025;16(1):41. 10.1186/s13287-024-04096-2. 39901212 10.1186/s13287-024-04096-2 PMC11792730

[6] Choudhery MS. Strategies to improve regenerative potential of mesenchymal stem cells. World J Stem Cells. 2021;13(12):1845–62. 10.4252/wjsc.v13.i12.1845. 35069986 10.4252/wjsc.v13.i12.1845 PMC8727227

